# The First Case Report of a Homozygous Consensus Acceptor Splice Variant in the NUP214 Gene Associated With Fetal Hydrops and Arthrogryposis Multiplex

**DOI:** 10.7759/cureus.73252

**Published:** 2024-11-07

**Authors:** Vasundhara Tamhankar, Smit J Patel, Tushar Kachhadiya, Shalin Vaniawala, Jayeshkumar Patel, Rajesh Bhammar, Shwetal Patel, Salil Vaniawala, Pramila Menon, Parag M Tamhankar

**Affiliations:** 1 Genetics, Centre for Medical Genetics, Mumbai, IND; 2 Genetics, SN GeneLab Pvt Ltd, Surat, IND; 3 Internal Medicine, Paaranu Women Super Specialties, Surat, IND; 4 Obstetrics and Gynaecology, Paaranu Women Super Specialties, Surat, IND; 5 Pediatrics, Dr. D.Y. Patil Medical College, Hospital and Research Centre, Dr. D.Y. Patil Vidyapeeth (Deemed to be University), Pune, IND

**Keywords:** arthrogryposis multiplex congenita, mrna export, nup214, protein network analysis, splice site mutation

## Abstract

The *NUP214* gene encodes a nuclear pore complex protein (nucleoporin, 214 kilodaltons) which plays a critical role in messenger RNA export to the cytoplasm and import of substrates from the cytoplasm. Biallelic mutations in the *NUP214* gene have been associated with susceptibility to acute infection-induced encephalopathy type 9 (ILAE9) (Online Mendelian Inheritance in Man (OMIM), 114350), an autosomal recessive disorder. Herein, we describe for the first time, a fetus with hydrops and arthrogryposis multiplex with a homozygous novel consensus splice site variant in the NUP214 gene, chr9:g.131127522A>G or c.46-2A>G (transcript ID NM_005085.4). Parents were heterozygous for the same variant. Mutations in either of 83 genes have been previously published to cause fetal arthrogryposis multiplex but mutations in *NUP214* have not been previously reported as per our search in the available medical literature (PubMed/MEDLINE (Medical Literature Analysis and Retrieval System Online) and Google Scholar). STRING (Search Tool for Retrieval of Interacting Genes/Proteins) analysis showed close interactions between *NUP214* and the other proteins GLE1, NUP88, NEK9, and THOC2. Thus, this case report expands the phenotype of *NUP214* gene-related human disease.

## Introduction

The *NUP214* gene encodes a nuclear pore complex protein (nucleoporin, 214 kilodaltons) which plays an important role in messenger RNA export to the cytoplasm and transport of substrates from the cytoplasm into the nucleus. Homozygous or compound heterozygous mutations in the *NUP214* gene have been associated with susceptibility to acute infection-induced encephalopathy type 9 (ILAE9) (Online Mendelian Inheritance in Man (OMIM), 114350), an autosomal recessive genetic disorder [1,2].

Fetal arthrogryposis multiplex (arthron = joint) is a condition that consists of contractures affecting two or more joints prenatally. Incidence is one in 3000 live births; however, the antenatal incidence may be higher implying high mortality. This syndrome is also known as fetal akinesia deformation sequence. Additional features include hydrops, intrauterine growth retardation, facial anomaly, skin pterygia, hypoplastic palmar dermal ridges, hypoplastic lungs, and polyhydramnios. Etiology can be primary due to mutations in chromosomes or genes or secondary due to extrinsic etiology leading to in-utero crowding such as oligohydramnios, uterine abnormalities, and twin pregnancies. Chromosomal disorders include trisomy 18 or trisomy 8. Single-gene etiologies consist of mutations in any of 83 genes [3,4]. 

This case report is the first description of a fetus with hydrops and arthrogryposis multiplex with a homozygous novel consensus splice site variant in the *NUP214* gene. Parents were heterozygous for the same variant. We have performed a medical literature search in available databases such as PubMed/MEDLINE (Medical Literature Analysis and Retrieval System Online) and Google Scholar for the terms *NUP214* gene and arthrogryposis and confirmed that mutations in the *NUP214* gene have not been previously reported in cases with fetal arthrogryposis. STRING (Search Tool for Retrieval of Interacting Genes/Proteins) analysis showed close interactions between *NUP214* and the other proteins *GLE1, NUP88, NEK9*, and *THOC2*. Mutations in the latter genes have been previously reported to be associated with fetal arthrogryposis.

## Case presentation

A third-degree consanguineous couple presented in their fifth pregnancy at 17 weeks gestational age. The mother had four previous first-trimester miscarriages which were not investigated. Karyotypes for the couple were normal. The antenatal ultrasound in the present pregnancy showed the fetus to have hydrops and arthrogryposis multiplex (Figures 1-9).

**Figure 1:**
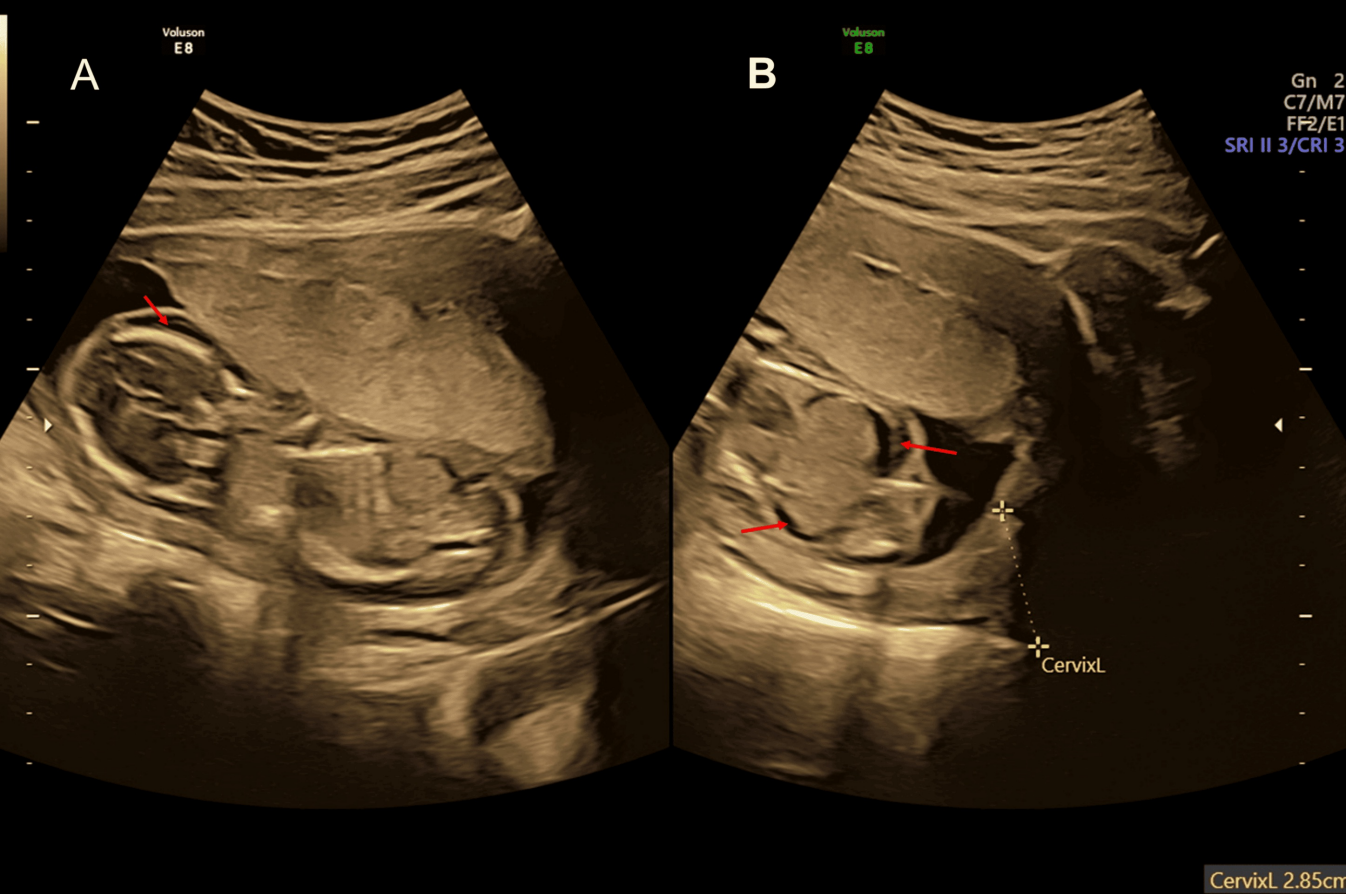
Antenatal ultrasound around the 17th week shows fetal hydrops and arthrogryposis; coronal section of the fetal body showing subcutaneous edema (red arrow). (A) head and trunk, (B) lower half of the body located near the cervix.

**Figure 2:**
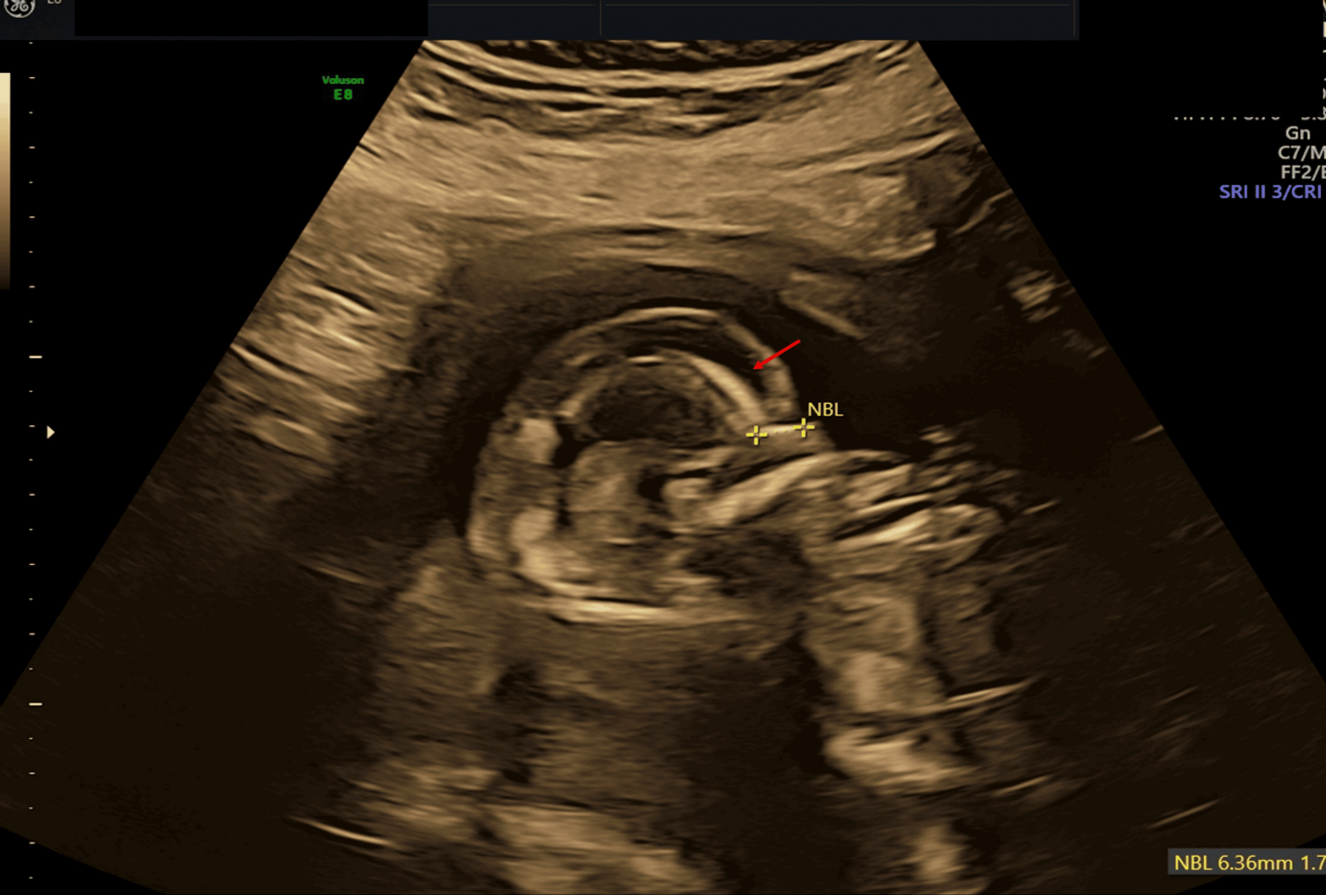
Antenatal ultrasound around the 17th week shows fetal hydrops and arthrogryposis; sagittal section of the fetal head and the face showing scalp edema (red arrow), nasal bone is present and normal NBL: nasal bone length

**Figure 3:**
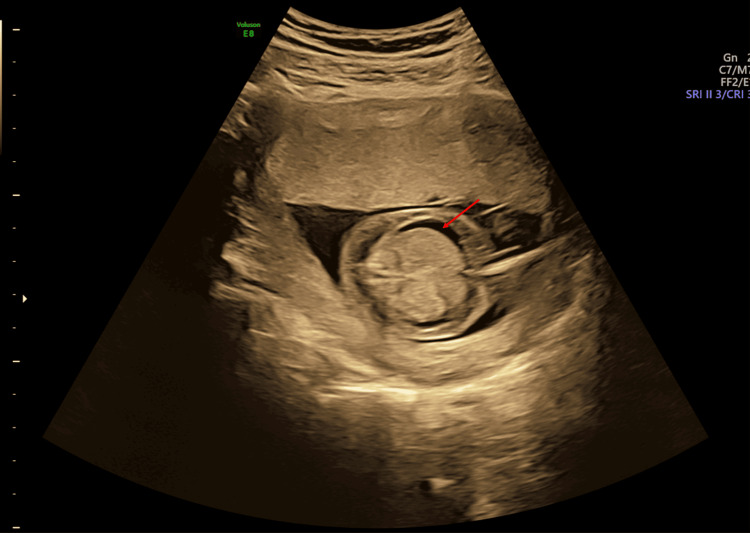
Antenatal ultrasound around the 17th week shows fetal hydrops and arthrogryposis; horizontal slice at level of the fetal abdomen showing fetal ascites (red arrow).

**Figure 4:**
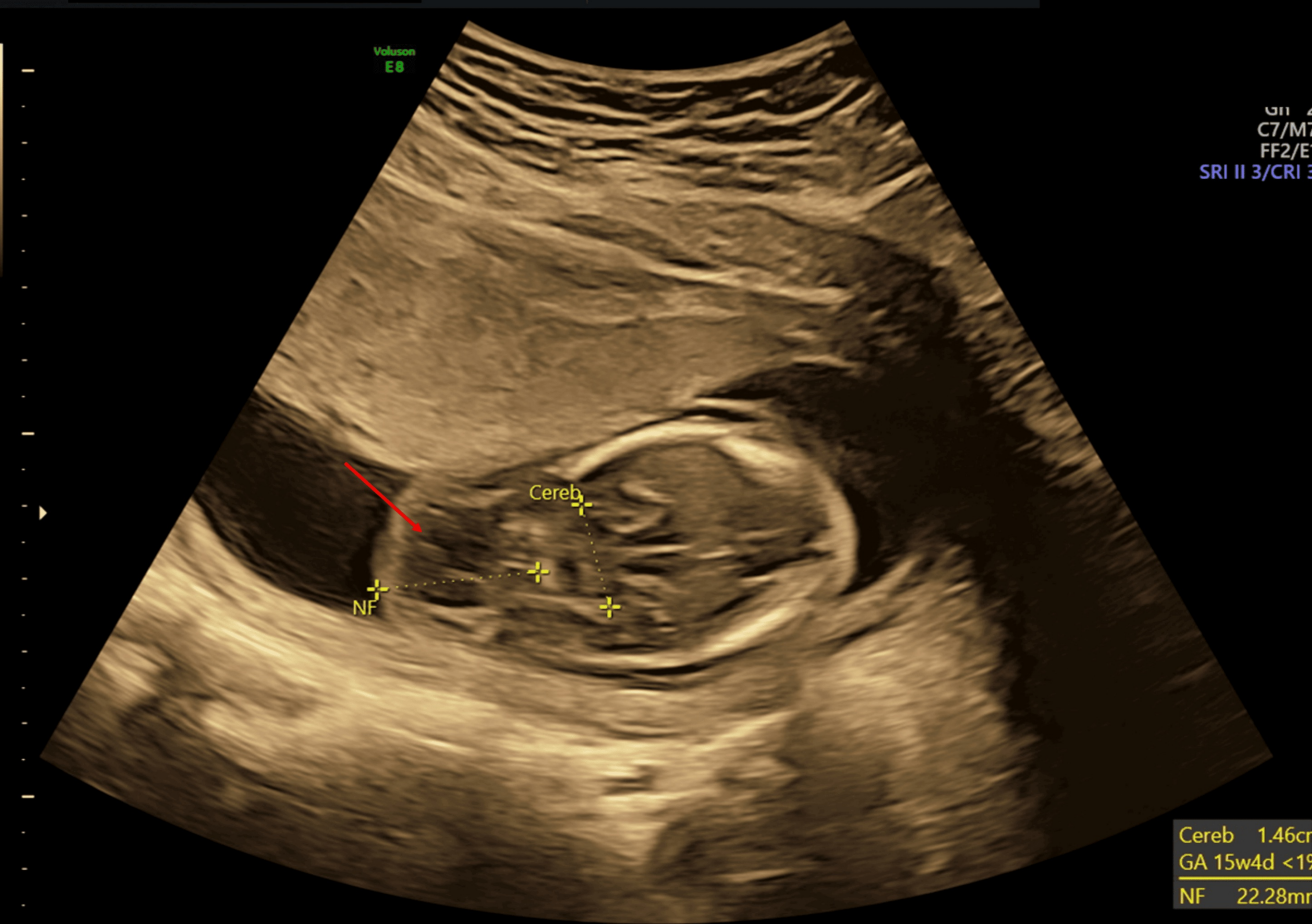
Antenatal ultrasound around the 17th week shows fetal hydrops and arthrogryposis; horizontal section of the fetal skull showing increased nuchal fold (red arrow).

**Figure 5:**
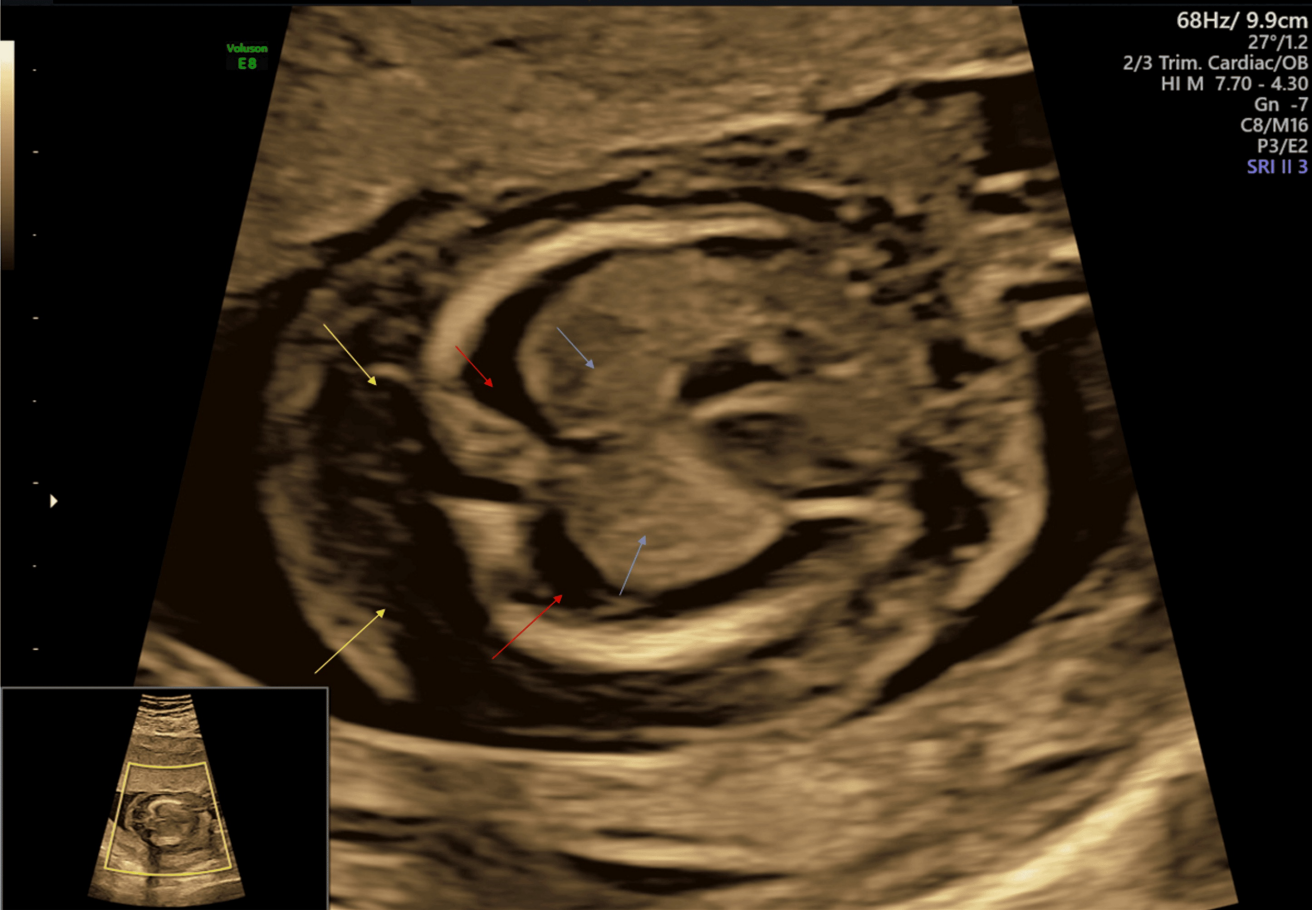
Antenatal ultrasound around the 17th week shows fetal hydrops and arthrogryposis; horizontal slice at level of fetal thorax showing lungs (blue arrows) surrounded by pleural effusion (red arrows) with subcutaneous edema (yellow arrow).

**Figure 6:**
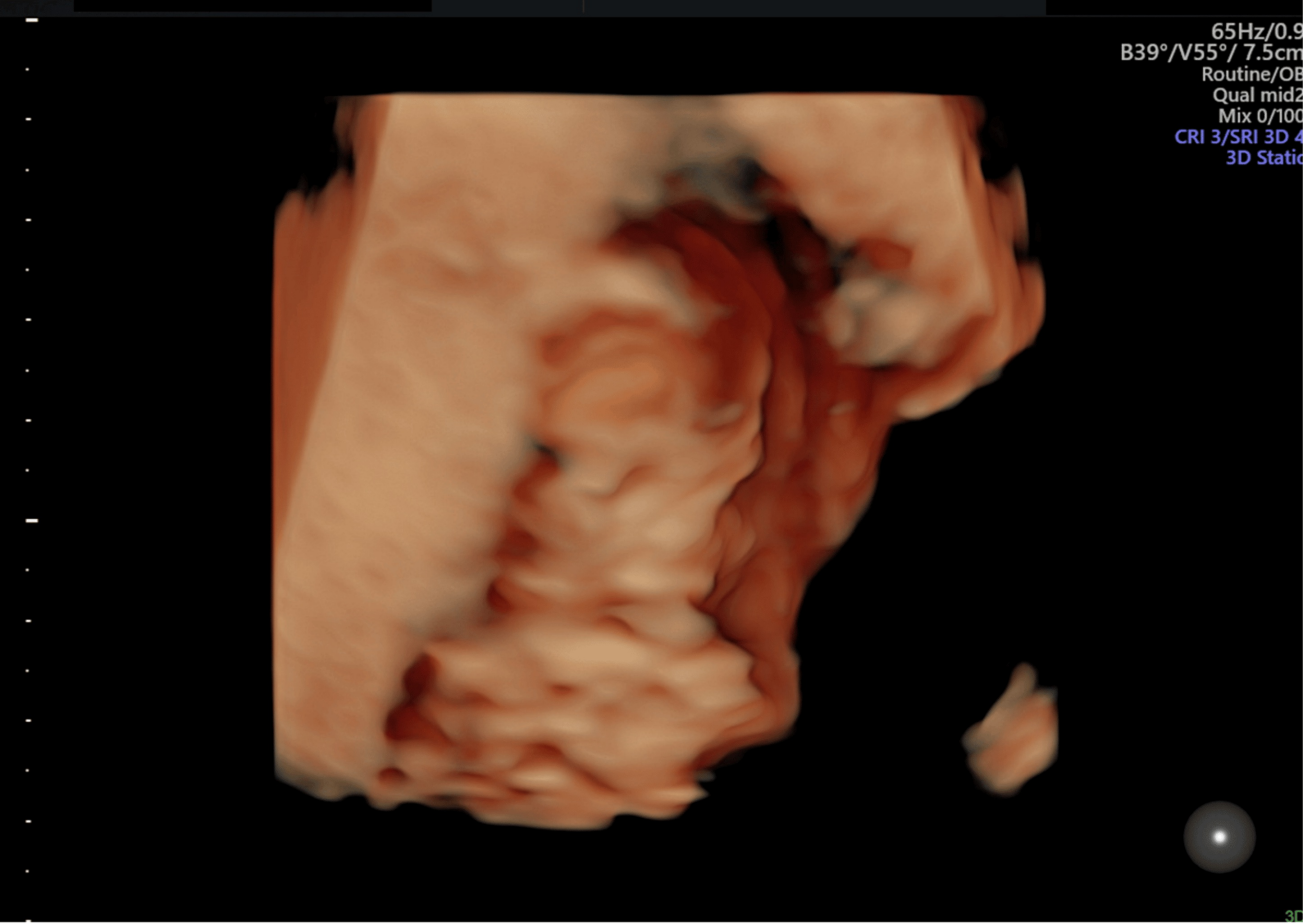
Antenatal ultrasound around the 17th week; three-dimensional rendered image of the fetal face shows normal features

**Figure 7:**
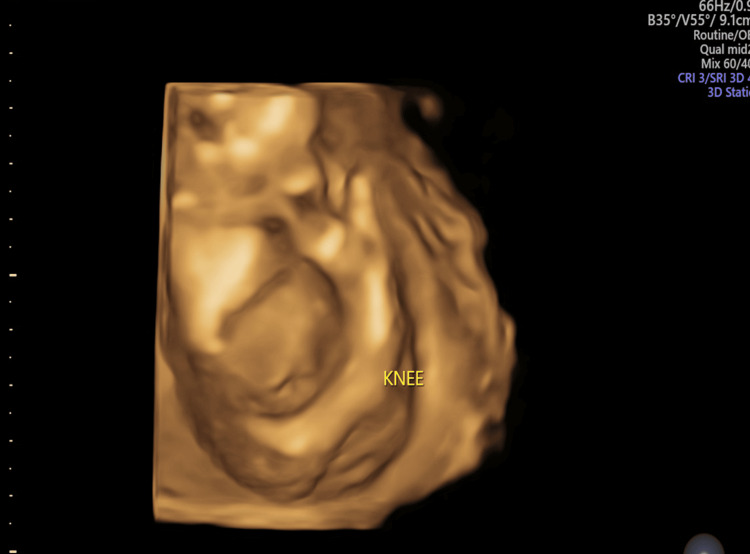
Antenatal ultrasound around the 17th week shows fetal hydrops and arthrogryposis; three-dimensional rendered image of the fetal lower limbs shows hyperextended knees

**Figure 8:**
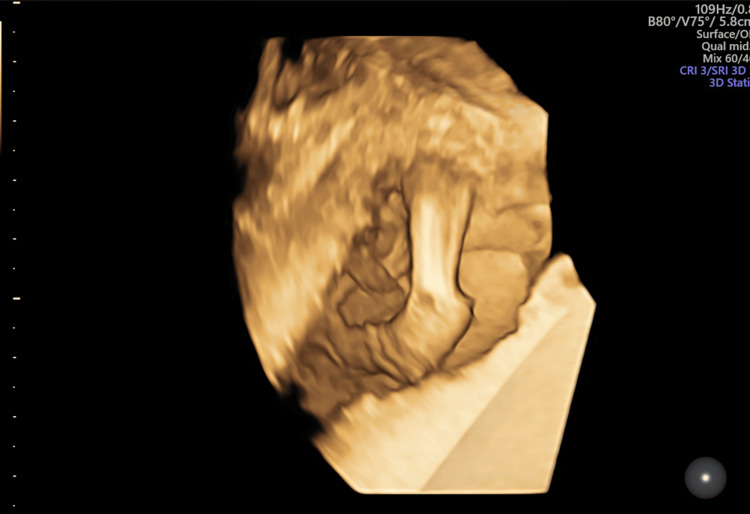
Antenatal ultrasound around the 17th week shows fetal hydrops and arthrogryposis; three-dimensional rendered image of the fetal leg and foot shows club foot

**Figure 9:**
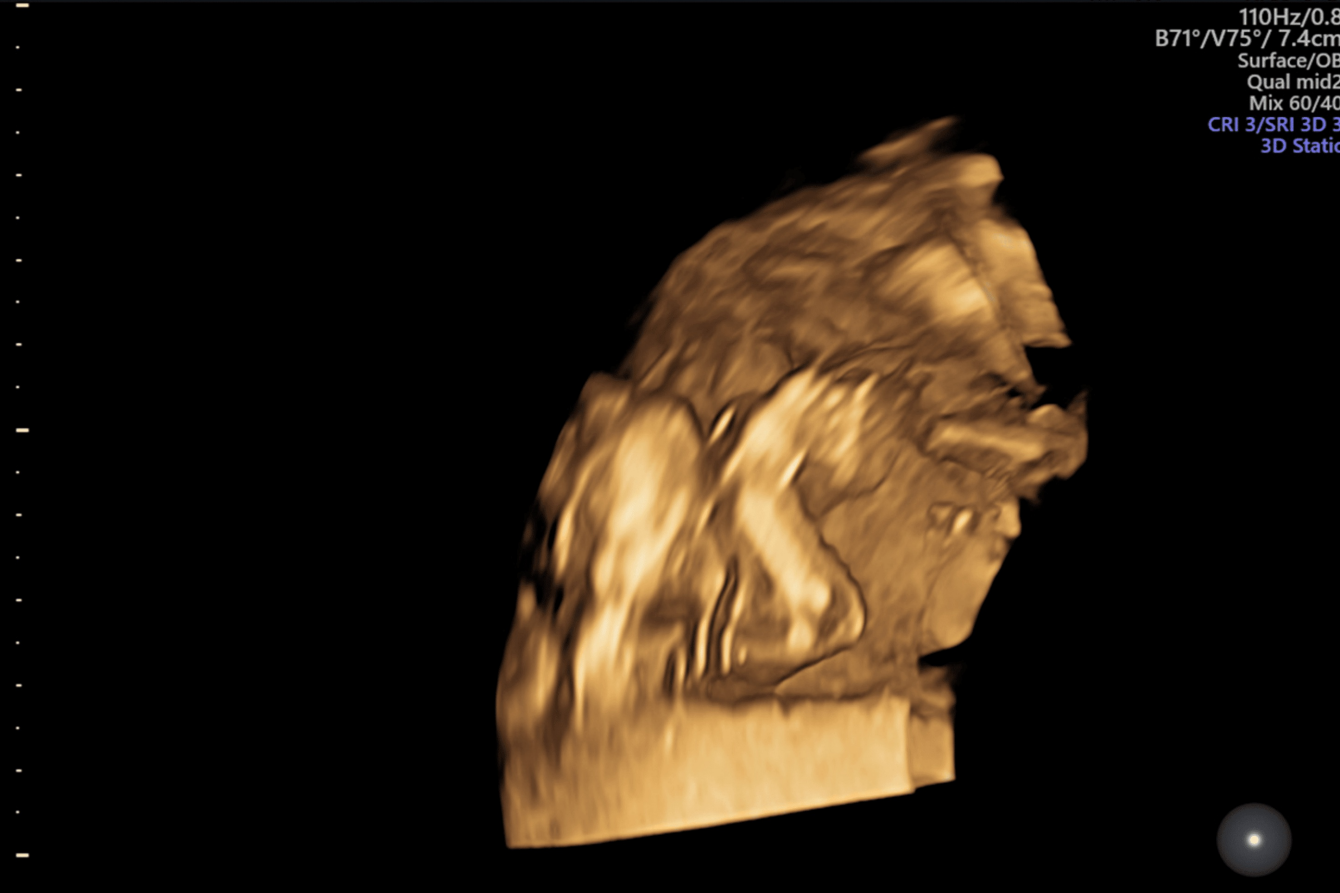
Antenatal ultrasound around the 17th week shows fetal hydrops and arthrogryposis; three-dimensional image showing fetal upper limbs in persistent flexion at the elbow.

The detailed ultrasound findings were as follows. The bi-parietal diameter was 33 mm (33rd centile for gestational age (GA)), head circumference was 123 mm (20th centile for GA), abdominal circumference was 142 mm (96th centile for GA), femur length was 16 mm (4th centile for GA), the cephalic index was 73 (dolichocephaly), foot length was 21 mm, the transcerebellar diameter was 15 mm (2nd centile), femur:foot length ratio was 0.76, nasal bone was 6.4 mm (92 centile), nuchal fold was increased (cystic hygroma) (22.3 mm). Midline falx was seen, both ventricles appeared normal, and no identifiable intracranial lesion was seen. Edema was present all around the skull and the body. The spine was visualized in longitudinal and transverse axes to be normal, and vertebrae and spinal canal appeared normal. Face as seen in coronal and profile views was normal. Orbits, nose, and mouth were normal. Thorax showed bilateral pleural effusion, and lungs were normal. The cardiac situs was normal and appeared to be in mid-position. Four chamber view was normal with symmetrical ventricles and symmetrical atria. Left and right ventricle outlet views, three-vessel view, and three-vessel trachea view were normal with normal size and arrangement of vessels with left-sided aortic and ductal arches with forward flow in both of them. The right subclavian artery appeared normal. Abdominal situs was normal. The stomach and bowel appeared normal. Moderate ascites was seen. The abdominal wall was intact. Both kidneys and the bladder appeared normal. All fetal long bones were visualized and appeared normal in length for the GA. Both upper limbs were persistently flexed at the shoulders and elbow with clenched fists. Both lower limbs showed persistent hyperextension at the knee. Bilateral club feet were present. The placenta was anterior and liquor was normal. Thus a diagnosis of hydrops fetalis and arthrogryposis multiplex was made. The couple opted for medical termination of pregnancy. The couple declined a fetal autopsy. However, after genetic counseling, they opted for genetic testing to identify the cause of the fetal anomalies.

**Figure 10:**
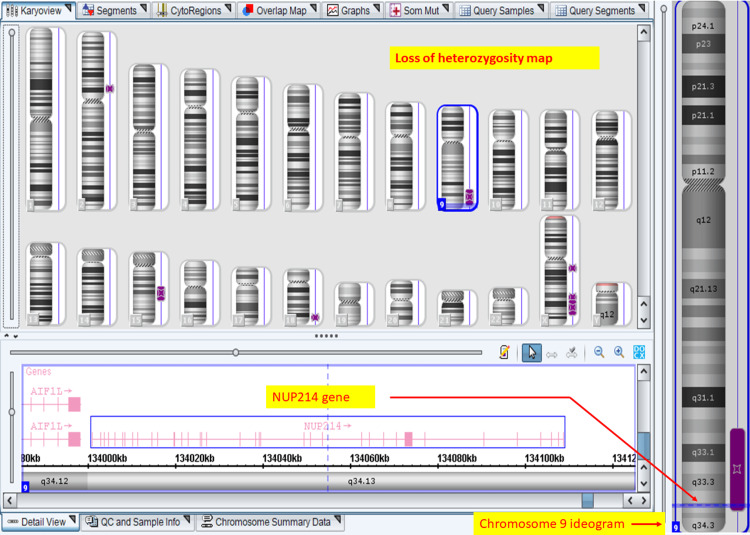
Fetal chromosomal microarray Virtual karyogram of fetal chromosomal microarray on CytoScan™ Optima (Thermo Fisher Scientific Inc., Waltham, Massachusetts, United States) 315K Chip shows a region of loss of heterozygosity (LOH) depicted by purple bands next to chromosome ideograms. LOH at chromosome 9q arm includes the *NUP214 *gene.

Chromosomal microarray performed on Applied Biosystems™ CytoScan™ Optima 315K chip (Thermo Fisher Scientific Inc., Waltham, Massachusetts, United States) did not reveal any significant aneuploidies or microdeletion-duplication syndromes. Significant loci of loss of heterozygosity (LOH) were present at the following sites: arr[GRCh37] 9q31.3q34.13 (113,690,929_135,410,052) (long arm of chromosome 9) and arr[GRCh37] 15q21.1q23 (48,654,125_68,786,285). The* NUP214* gene (chromosome locus 9q34.13) is present in the 21.7 megabases region of LOH on chromosome 9 (Figure 10). 

**Figure 11:**
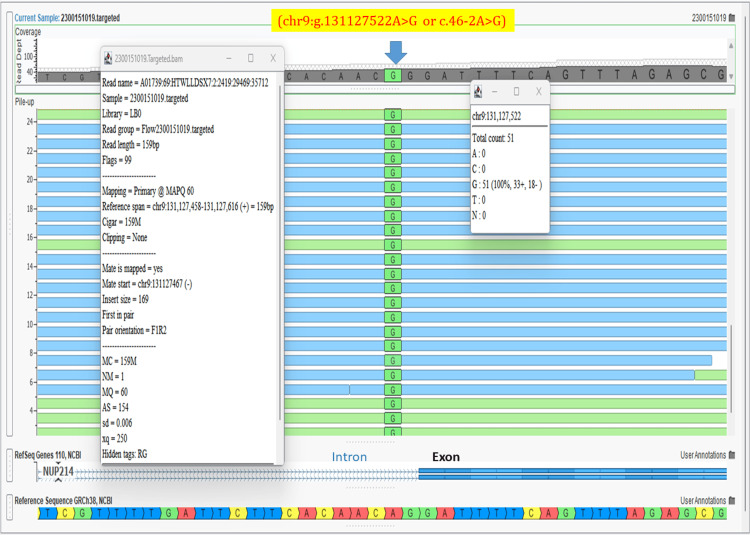
Homozygous splice site variant in the NUP214 gene Screenshot of the Integrated Genomic Viewer (VarSeq; Golden Helix Inc., Bozeman, Montana, United States) of the binary alignment map (BAM) file of the whole exome sequence data for the affected fetus shows homozygous mutation chr9:g.131127522A>G or c.46-2A>G in *NUP214* gene

A whole exome analysis (20321 genes with 37 mitochondrial genes) (Twist exome panel 2.0 with additional mitochondrial gene coverage added; Twist Biosciences Inc., South San Francisco, California, United States) was performed for the fetus (postnatally collected fetal skin tissue). In addition, to single nucleotide variants and small insertion-deletions, copy number variants were detected from the targeted sequence data using ExomeDepth (version 1.1.10) and this bioinformatics pipeline has been clinically validated [5]. This lab has been accredited by the National Accreditation Board for Testing and Calibrating Laboratories (NABL) of India. The fetus was identified to be homozygous for a novel variant in the *NUP214 *gene with genomic coordinates as chr9:g.131127522A>G (GRCh38 format) or c.46-2A>G (transcript ID NM_005085.4) (read depth: 51x) (located in Intron 1) and affecting the consensus acceptor splice site between intron 1 and exon 2 (Figure 11).

**Figure 12:**
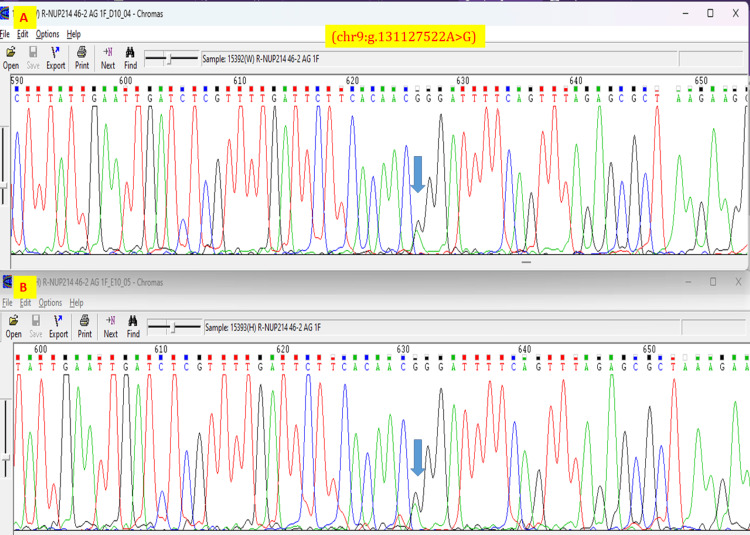
Sanger sequence chromatograms for the NUP214 gene for the couple: (A) mother and (B) father Sanger sequencing chromatograms for the couple show heterozygosity for the *NUP214* gene variant chr9:g.131127522A>G (blue arrow)

This variant is absent in gnomAD, 1000Genomes, and ClinVar public databases. The variant is likely pathogenic according to the American College of Medical Genetics pathogenicity criteria satisfying PVS1 (pathogenic very strong criteria 1: null variant in a gene where loss of function is a known mechanism of disease) and PM2 (pathogenic moderate criteria 2: extremely low frequency in gnomAD database) [6]. Exome copy number variant analysis did not reveal any significant chromosomal microdeletions/ microduplications. The couple was identified to be heterozygous for the same variant in the *NUP214* gene by targeted Sanger sequencing of 700 nucleotides surrounding this region (Figure 12).

The couple had normal clinical examination findings.

## Discussion

The *NUP214* gene encodes nucleoporin 214 kilodalton protein, an essential component of the nuclear pore complex (NPC) through which nucleocytoplasmic transport occurs. The NPC exports mRNA to the cytoplasm for translation into various proteins. The nucleoporin 214 kilodalton protein in a complex with other proteins forms an annual ring at the cytoplasmic aspect of the NPC that anchors the cytoskeleton to the NPC. The other members of the NPC proteins include NUP98, NUP62, NUP54, NUP58/45, NLP1, and NUP153 [1].

*NUP214* gene mutation

Mutations in* the NUP214 *gene are known to lead to susceptibility to acute infection-induced encephalopathy type 9 (ILAE9) (Online Mendelian Inheritance in Man (OMIM), 114350, an autosomal recessive genetic disorder). Shamsheldin et al. identified homozygous missense variant p.D154G (exon 4) in the *NUP214* gene in three sisters from Saudi [1]. They presented in early childhood with recurrent admissions for fever with seizures, hyponatremia, and encephalopathy which resulted in progressive neurodegeneration, microcephaly, and brain atrophy. Fichtman et al. reported the homozygous p.R38C (exon 2) mutation in *the NUP214* gene of two affected members from Palestine and compound heterozygous mutations p.P387S (exon 11) and p.P525LfsX6 (exon 12) in two sisters from northern Europe [2]. These patients had recurrent episodes of neurological symptoms such as seizures, ataxia, involuntary movements, encephalopathy triggered by febrile illnesses, and progressive neuro-regression with cerebral and cerebellar atrophy. None of these patients were reported to have fetal presentation such as hydrops or arthrogryposis. Fichtman et al. showed that these mutations led to a 50% decrease in NUP214 and NUP88 levels compared to controls and led to decreased rim localization of these proteins [2]. Functional analysis showed decreased but not abolished (hypomorphic rather than amorphic) protein import and mRNA export activities in the nuclear pores. They found plugs in the nuclear pores which probably represented mRNA. These abnormalities could be partially rescued by transfection of wildtype NUP214 and NUP88 [2]. 

**Figure 13:**
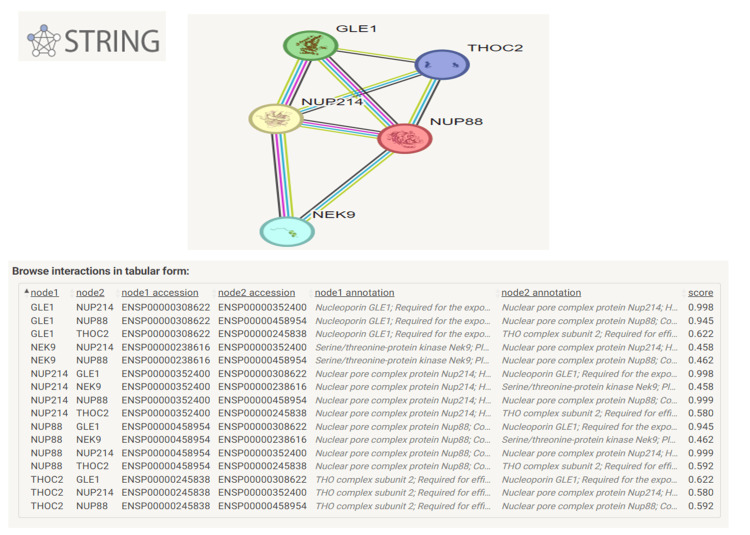
STRING database analysis for the protein interactions between NUP214 and THOC2, GLE1, NUP88, and NEK9 STRING database analysis for protein interactions between *NUP214 and THOC2, GLE1, NUP88* and *NEK9* (genes known to be associated with arthrogryposis) show significant protein-protein interactions. The blue interconnecting line indicates known interactions from curated databases; the purple line: experimentally determined interaction, the light green line: interactions from text-mining of published literature, and the black line: co-expression. The interactions are scored from zero (minimum) to one (highest). STRING: Search Tool for Retrieval of Interacting Genes/Proteins

The severity of presentation in our case could be attributed to the c.46-2A>G being probably an amorph (near complete/complete loss of activity) rather than a hypomorph, it is the most proximal (to the N-terminal of the protein) *NUP214* gene variant identified till date. The limitation of our study is that we could not do RNA or protein studies. STRING analysis (https://string-db.org) of 83 genes known to be associated with arthrogryposis with *NUP214 (ACTA1, ADCY6, ADGRG6, AGRN, BIN1, CACNA1E, CASK, CFL2, CHAT, CHRNA1, CHRNB1, CHRND, CHRNE, CHRNG, CHST14, CHUK, CNTNAP1, COL6A2, COLQ, DHCR24, DNM2, DOK7, DPAGT1, ECEL1, EGR2, ERBB3, ERCC5, ERCC6, EXOSC3, FBN2, FHL1, FKBP10, FKTN, FLVCR2, GBA, GBE1, GFPT1, GLDN, GLE1, KAT6B, KIAA1109, KLHL40, LGI4, LMNA, MPZ, MTM1, MUSK, MYBPC1, MYH2, MYH3, MYH8, NALCN, NEB, NEK9, NUP188, NUP214, PIEZO2, PLOD2, PMM2, PPP3CA, RAPSN, RARS2, RIPK4, SCO2, SELENON, SMN1, TGFB3, THOC2, TK2, TNNI2, TNNT1, TNNT3, TPM2, TPM3, TRPV4, TSEN2, TSEN54, UBA1, VIPAS39, VPS33B, VRK1, ZBTB42, ZC4H2*) showed close interactions between NUP214 and proteins GLE1, NUP88, THOC2 and NEK9, mutations in whom have been previously reported to cause fetal arthrogryposis. Figure 13 shows selected STRING analysis for NUP214, GLE1, NUP88, THOC2, NEK9 proteins.

**Figure 14:**
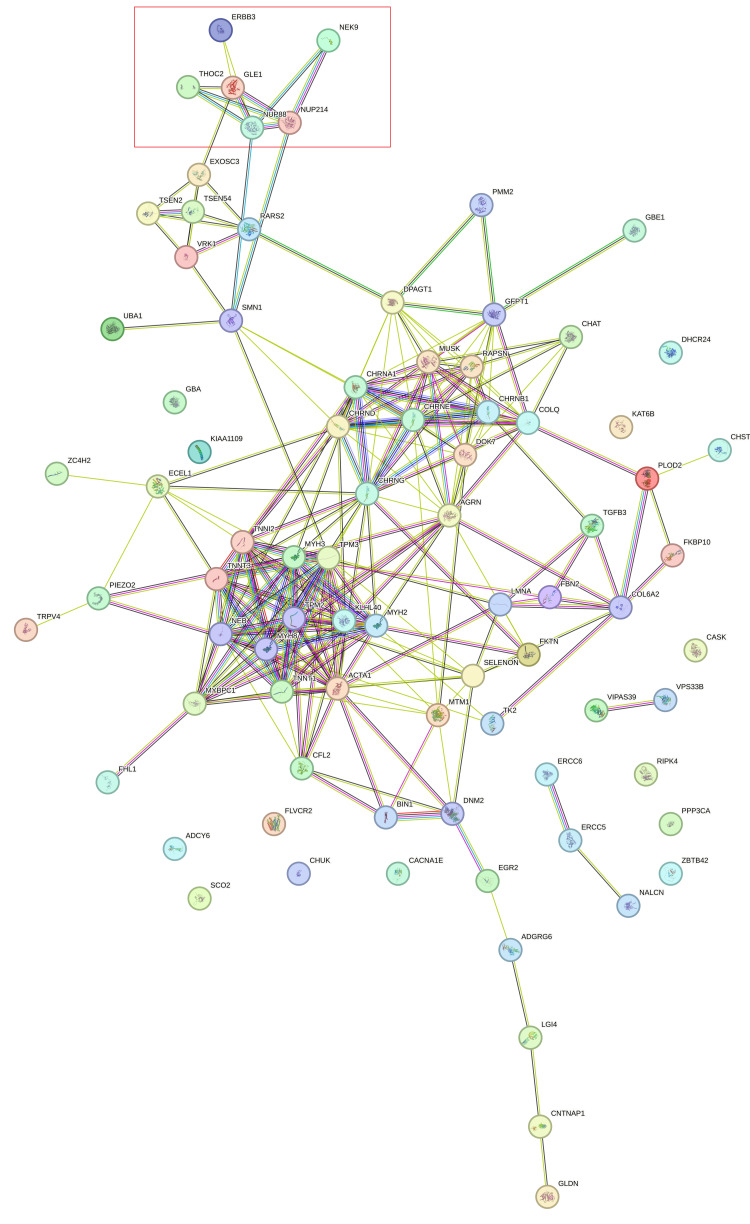
STRING database analysis for protein interactions between NUP214 and 83 genes STRING database analysis for protein interactions between NUP214 and 83 genes included in the list of genes causative for arthrogryposis. The red box includes NUP214 and the following closely related proteins: NUP88, GLE1, THOC2, and NEK9 STRING: Search Tool for Retrieval of Interacting Genes/Proteins

Figure 14 shows STRING protein network analysis for all 83 genes and *NUP214*.

Closely associated genes related to NUP214 (viz. *GLE1, NUP88, THOC2, NEK9*) and the causation of arthrogryposis

Mutation in the *NUP88* gene (nucleoporin, 88 kilodaltons) leads to fetal akinesia deformation sequence 4 (FADS) which in addition to arthrogryposis, includes hydrops, polyhydramnios, and facial dysmorphism and leads to miscarriage or perinatal death. Bonnin et al. identified recessive loss of function variants in the *NUP88* gene namely p.D434Y, p.R509X, p.E634del in these patients [7]. Griffis et al. had previously shown that the NUP88 protein resides on both the cytoplasmic and the nuclear side of nuclear pore complexes [8]. On the cytoplasmic side, it associates with NUP214, NUP62, and NUP98 proteins, whereas on the nuclear side, it binds the intermediate filament protein LMNA. Bernad et al. showed that the NUP88-NUP214 complex plays a crucial role in the export of proteins and pre-ribosomes which is mediated by exportin 1 (XPO1) [9]. 

Mutation in the *GLE1* gene (GLE1 RNA export mediator) leads to lethal congenital contracture syndrome type 1 (LCCS1) and congenital arthrogryposis with anterior horn cell disease (OMIM). Both these have overlapping phenotypes that occur in utero or in the perinatal period. In addition to arthrogryposis, other features include pulmonary hypoplasia, facial dysmorphism, motor neuron (anterior horn cell), axon loss, and hydrops. The GLE1 protein regulates gene expression at multiple steps, namely mRNA export, translation initiation, and translation termination [10]. Nousianen et al. found a founder mutation in the *GLE1* gene in Finnish cases with LCCS1, namely c.432-10A>G (referred to as FinMajor mutation) [11]. 

Tamhankar et al. were the first to identify the association between *THOC2* gene mutation and arthrogryposis in an Indian family [12]. A novel splice site mutation c.2482-1_2484delGTCA in the *THOC2* gene was found in the hemizygous state in two male fetuses with arthrogryposis multiplex. The same variant was also identified by Dubucs et al. in a fetus with arthrogryposis from a French family [13]. Additional abnormalities noted were hydrops, hypoplasia of the cervical vertebral bodies, lack of ossification of the pubic rami, absence of the talus, and presence of microscopic cytoplasmic bodies in the muscle fibers. The *THOC2* gene encodes the largest subunit of the highly conserved TREX (Transcription-Export) mRNA export pathway [13]. 

Mutation in the *NEK9* (Nima-related kinase-9) gene leads to lethal congenital contracture syndrome type 10 (LCCS10) which in addition to arthrogryposis includes short limbs (bowed femora), narrow thorax, and perinatal death. Casey et al. identified the p.R497X mutation in the *NEK9* gene in two unrelated Irish Traveller families [14]. The *NEK9* gene is a regulator of mitosis, participating in the control of spindle movement and chromosome separation [15].

## Conclusions

We described a fetus with hydrops and arthrogryposis with homozygous recessive consensus splice site variant c.46-2A>G in the *NUP214* gene. This is the most proximal (to the N terminal of the protein) *NUP214* gene variant detected to date, hence probably an amorph variant (complete loss of function) as compared to previously published hypomorph variants (partial loss of function). Homozygosity mapping by microarray and STRING protein-protein network analysis, and medical literature of related/ interacting genes such as *NUP88, GLE1, THOC2* (these three along with the *NUP214* gene are involved in mRNA export pathway), and *NEK9*, suggested that the *NUP214* gene variant c.46-2A>G could be possibly causal for arthrogryposis in the present case. Our case report expands on the phenotype of *NUP214* gene-related disease. Further research is required to elucidate the mechanism of production of arthrogryposis by *NUP214* gene variants.
